# Tumor‐derived exosomal PD-L1: a new perspective in PD-1/PD-L1 therapy for lung cancer

**DOI:** 10.3389/fimmu.2024.1342728

**Published:** 2024-03-18

**Authors:** Yunjiao Wu, Huichao Fu, Jingwei Hao, Zhaoyang Yang, Xinyi Qiao, Yingjie Li, Rui Zhao, Tie Lin, Yicun Wang, Meng Wang

**Affiliations:** ^1^ Department of Respiratory Medical Oncology, Harbin Medical University Cancer Hospital, Heilongjiang, Harbin, China; ^2^ Department of Orthopedic Surgery, The Second Affiliated Hospital of Harbin Medical University, Heilongjiang, Harbin, China; ^3^ Department of Surgery, The First Affiliated Hospital of Harbin Medical University, Heilongjiang, Harbin, China; ^4^ Department of Medical Research Center, Second Hospital of Jilin University, Jilin, Changchun, China

**Keywords:** lung cancer, exosomes, PD-L1, immune escape, immunotherapy

## Abstract

Exosomes play a crucial role in facilitating intercellular communication within organisms. Emerging evidence indicates that a distinct variant of programmed cell death ligand-1 (PD-L1), found on the surface of exosomes, may be responsible for orchestrating systemic immunosuppression that counteracts the efficacy of anti-programmed death-1 (PD-1) checkpoint therapy. Specifically, the presence of PD-L1 on exosomes enables them to selectively target PD-1 on the surface of CD8+ T cells, leading to T cell apoptosis and impeding T cell activation or proliferation. This mechanism allows tumor cells to evade immune pressure during the effector stage. Furthermore, the quantification of exosomal PD-L1 has the potential to serve as an indicator of the dynamic interplay between tumors and immune cells, thereby suggesting the promising utility of exosomes as biomarkers for both cancer diagnosis and PD-1/PD-L1 inhibitor therapy. The emergence of exosomal PD-L1 inhibitors as a viable approach for anti-tumor treatment has garnered significant attention. Depleting exosomal PD-L1 may serve as an effective adjunct therapy to mitigate systemic immunosuppression. This review aims to elucidate recent insights into the role of exosomal PD-L1 in the field of immune oncology, emphasizing its potential as a diagnostic, prognostic, and therapeutic tool in lung cancer.

## Introduction

The utilization of immune checkpoint inhibitors (ICIs) presents a hopeful strategy for managing advanced lung cancer due to their ability to enhance the CD8+ T cell-mediated response, which includes direct cytotoxic activity against tumor cells (1). Tumor cells express programmed cell death ligand-1 (PD-L1), which interacts with programmed death-1 (PD-1) on the T cell, counteracting the T cell receptor (TCR) signaling cascade by Src homology-2 domain-containing phosphatase 2 (SHP2) (2). This interaction effectively inhibits the T cell activation signaling pathway, including the rat sarcoma (Ras)/mitogen-activated extracellular signal-regulated kinase (MEK)/extracellular regulated protein kinases (ERK) pathway and the phosphoinositide 3-kinase (PI3K)/protein kinase B (AKT)/mammalian target of rapamycin (mTOR) pathway. It dampens the tumor’s immune response (3). The current study predominantly attributes this immunosuppression to membrane PD-L1 (mPD-L1) (4).

**Figure 1:**
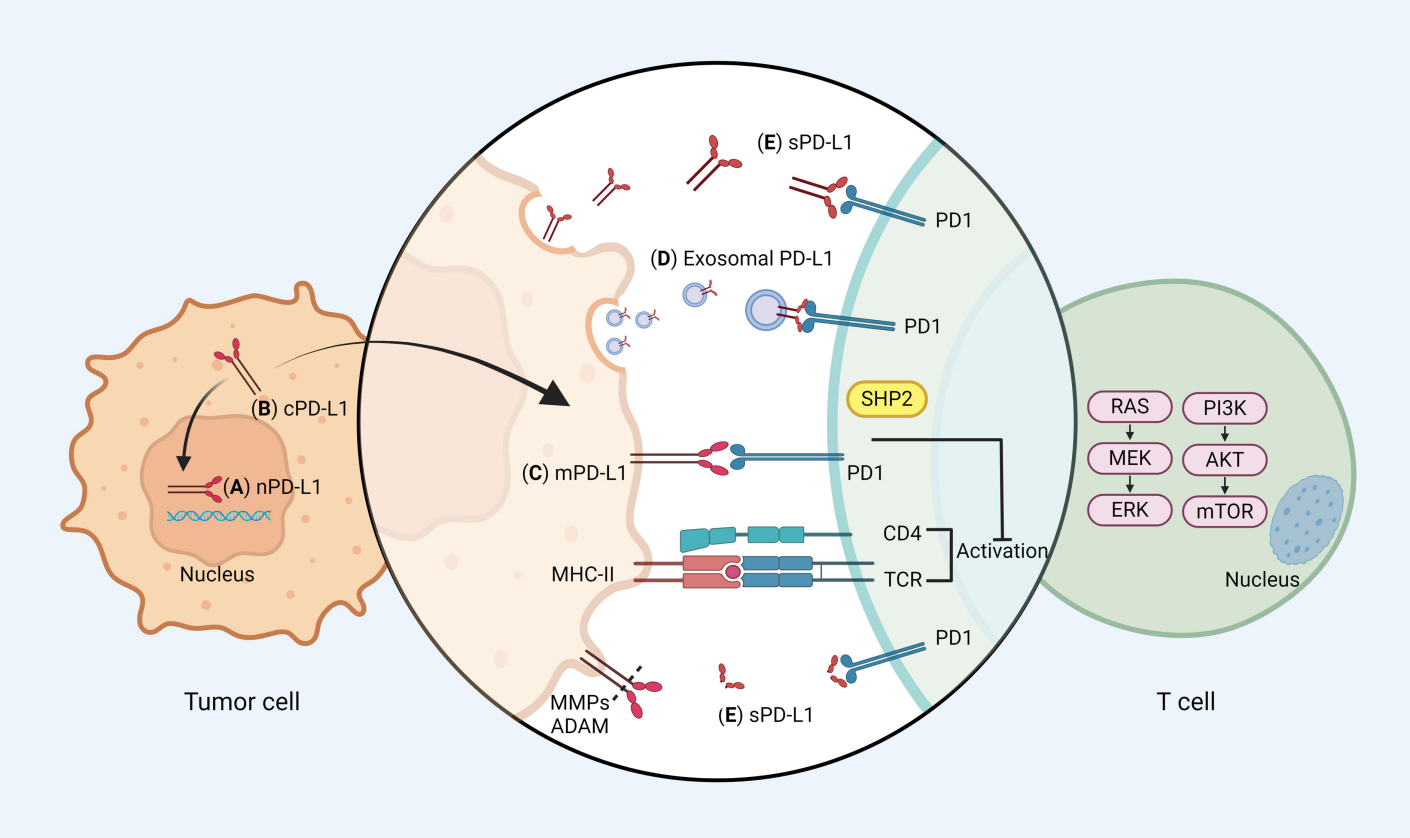
PD-1/PD-L1 signaling pathways. **(A)** Nuclear PD-L1 (nPD-L1): nPD-L1 is located in the nucleus and may be associated with enhanced chemotherapy resistance. **(B)** Cytoplasmic PD-L1 (cPD-L1): cPD-L1 is located in cytoplasm, and potentiates to transfer to mPD-L1. **(C)** Membrane PD-L1 (mPD-L1): the classical function of PD-L1, which is mainly mediated by mPD-L1. mPD-L1 is located on tumor cell membranes and can bind to PD-1 on T cells, recruiting the Src homology 2 domain containing phosphatases 2 (SHP2) to the PD-1 cytoplasmic domain, which dephosphorylates signaling molecules of the phosphoinositide 3-kinase (PI3K)/protein kinase B (AKT) and mitogen activated protein kinases (MAPK) pathways, thereby restricting T cell proliferation, activation, and survival. **(D)** Exosomal PD-L1: exosomal PD-L1 has the same membrane topology as mPD-L1, which causes tumor progression mainly by inhibiting T cell activation. **(E)** Soluble PD-L1 (sPD-L1): sPD-L1 generated from either endogenous secretion or cleaved fraction of mPD-L1. sPD-L1, like mPD-L1, binds to PD-1 to transmit negative regulatory signals. Different forms of PD-L1 can be recycled between the cytoplasm and the membrane. mPD-L1 enters the cell membrane via huntingtin interacting protein 1-related (HIP1R), whereas cPD-L1 is recycled to the cell membrane via transporter protein particle subunit 4 (TRAPPC4). Figure created with BioRender.com.

Beyond the mPD-L1, more forms of PD-L1 have been identified, encompassing nuclear PD-L1 (nPD-L1), cytoplasmic PD-L1 (cPD-L1), soluble PD-L1 (sPD-L1), and exosomal PD-L1. This diversity underscores the intricate roles of PD-L1 in tumorigenesis, spanning both immune and non-immune functionalities (5) (**Figure 1**). nPD-L1 markedly escalates the expression of genes pertinent to immune and pro-inflammatory pathways within tumor cells, thereby catalyzing tumor progression (6). cPD-L1 predominantly modulates tumor cell proliferation, apoptosis, and resistance to chemotherapeutic and radiological interventions (7). cPD-L1 was found to inhibit the degradation of mRNA molecules associated with DNA damage, thereby attenuating DNA damage induced apoptosis. sPD-L1, paralleling the function of mPD-L1, predominantly binds to PD-1 to propagate inhibitory signaling, the exact mechanism needs to be further elucidated (8). Exosomal PD-L1 significantly impedes lymphocyte activity and migrates to PD-L1-negative tumor cells and immune cells, instigating both localized and systemic immunosuppression, thereby facilitating tumor growth and proliferation (9).

Intriguingly, there are dynamic interactions between these different PD-L1 forms. cPD-L1 can be transported to the cell surface by trafficking protein particle complex (TRAPPC4) cycle to replenish mPD-L1 recognized by antibodies, while mPD-L1 can internalize into the cytoplasm and transform into cPD-L1 (10, 11). This dynamic exchange might elucidate the partial ineffectiveness of antibody blockade therapies because when blocking antibodies are degraded and eliminated by protein hydrolysis or systemic clearance, intracellular PD-L1 has the potential to migrate to the cell surface and regain its immune evasive capacity.

A growing number of researchers consider that exosomal PD-L1 plays a role in a novel mechanism that helps tumors evade the immune system, and it has been recognized as a developing field of immunotherapy for lung cancer. Blocking exosomal secretion and immune checkpoint proteins may augment the efficacy of anti-cancer immune reactions and open up new prospects in tumor immunotherapy (12, 13). It is worth noting that exosomes obtained from healthy cells and cancer cells exhibit significant variations in quantity and composition, indicating the presence of specificity to some degree (14). Consequently, the detection of exosomes holds potential value for the early diagnosis and prognostic assessment of lung cancer. Exploring the potential clinical uses of exosomal PD-L1 as both a biomarker for tumors and a target for therapy is of great importance.

In this review, we focus attention on the role of exosomal PD-L1 in the modulation of the immune system. In addition, we emphasize the potential application of exosomal PD-L1 in lung cancer immunotherapy.

## What are exosomes and exosomal PD-L1?

**Figure 2:**
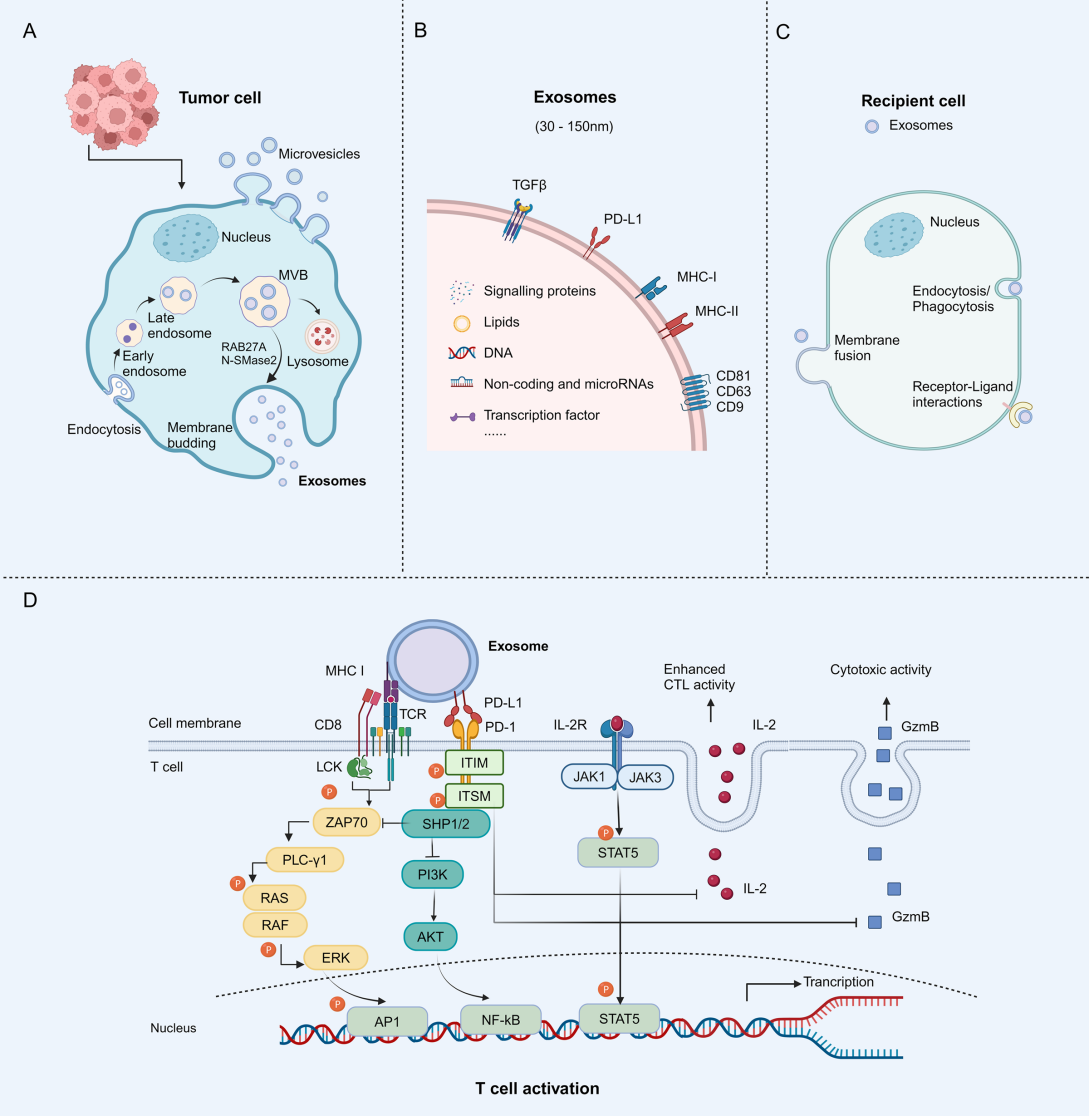
Biological characteristics of tumor-derived exosomes (TEXs) and mechanisms of exosomal PD-L1 to inhibit T cell activity. **(A)** Tumor cells contain TEXs. The process begins with membrane invagination, followed by the production of endosomes, the formation of intraluminal vesicles (ILVs), and the accumulation of multivesicular bodies (MVBs). These MVBs either bind to the plasma membrane and become exosomes or are degraded by lysosomes. Neutral sphingomyelinase 2 (nSMase2) and Rab27A proteins regulate exosomes secretion. **(B)** The exosomes contain specific payloads, such as proteins, DNAs, lipids, miRNAs, and others. Specific membrane proteins that act as biomarkers, such as PD-L1, major histocompatibility complex (MHC) I and II, as well as some cytoskeletal proteins, are present on the surface of exosomes. **(C)** The exosomes ultimately release their contents into recipient cells through different mechanisms, including endocytosis/phagocytosis, direct membrane fusion, and receptor-ligand interactions. **(D)** Mechanisms of exosomal PD-L1 to inhibit T cell activity. The interaction between PD-L1 and PD-1 induces phosphorylation of the cytoplasmic immunoreceptor tyrosine based inhibitory motif (ITIM) and the immunoreceptor tyrosine based switch motif (ITSM). Phosphorylated ITIM and ITSM recruit SHP-1 and SHP-2 protein tyrosine phosphatases to attenuate T cell activation signals. Granzyme B (GzmB) is an effector molecule with cytotoxic activity in T cells, whereas interleukin-2 (IL-2) enhances the activation and survival of cytotoxic T lymphocytes (CTL) through the janus kinase (JAK)/signal transducer and activator of transcription (STAT) pathway. Exosomal PD-L1 inhibits CD8+ T cell proliferation, cytokine production, and cytotoxicity by suppressing GzmB expression and inhibiting IL-2 production. Figure created with BioRender.com.

Extracellular vesicles (EVs) are the term for particles that are delimited by a lipid bilayer and cannot replicate on their own (vesicular component of extracellular particles) (15). EVs carry various biological macromolecule ingredients including DNA, RNA, proteins, and lipids, which are released into the extracellular environment, transfer cargo to recipient cells, and thus play important roles in cell-to-cell communication. EVs are generally split into three subtypes based on their mechanism of biogenesis: exosomes, microvesicles (MVs), and apoptotic bodies (16). Almost all kinds of normal or tumor cells secrete exosomes, which are nanovesicles with a lipid bilayer and are biologically active (17–20). Exosomes originate from the endocytosis pathway and bud inward from vesicles in the late endosome to form a multivesicular body (MVB). The MVBs merge with the cell membrane and the vesicles are ultimately released through exocytosis as exosomes measuring 30-150 nm (21). Exosome secretion is regulated by Rab proteins and neutral sphingomyelinase 2 (nSMase2), while the endosomal sorting complex required for transport (ESCRT) participates in the packaging of biomolecules into exosomes (22). Several researchers have discovered that exosomes transport immune checkpoint proteins like PD-L1, cytotoxic T lymphocyte associated protein 4 (CTLA-4), and T cell immunoglobulin domain and mucin domain-3 (TIM-3) (17, 23–26). The presence of PD-L1 on the surface of tumor-derived exosomes (TEX) contributes to tumor infiltration, metastasis, and immune evasion (27) (**Figures 2A–C**). Several cytokines, such as transforming growth factor-β (TGF-β) and interferon-γ (IFN-γ), have also been found to promote exosomal PD-L1 production (28, 29).

Exosomal PD-L1 can also be produced by other types of cells. In melanoma, macrophages inside tumor tissues, induced by the tumor microenvironment, turn into tumor-associated macrophages (TAM) and produce exosomes bearing PD-L1, which act on activated CD8+ T cells to inhibit T cell proliferation and tumor-killing effect (30). In addition to exosomal PD-L1, Chen et al. have shown that MVs from tumor cells also contained membrane-associated PD-L1 and could compete with tumor cells to bind anti-PD-L1 drugs (31). Glioblastoma (GBM)-associated regulatory B cells (Breg) were characterized by immunosuppressive activity toward activated CD8+ T cells. Lee-Chang et al. found that GBM-associated myeloid-derived suppressor cells (MDSCs) promoted Bregs function by delivering MVs transporting membrane-bound PD-L1 (32). These studies demonstrate the heterogeneity of EV types carrying PD-L1. Although MVs and exosomes are structurally similar, they differ in size, lipid composition, content, and cellular origin (33). Those PD-L1-bearing extracellular vesicles play an integral role in the formation of the immunosuppressive microenvironment, and their specific biological functions need to be further explored. According to the conventional wisdom, PD-L1-mediated immunosuppression was mostly based on the direct physical contact between tumor cells and tumor-infiltrating T cells. The mechanism of tumor immunosuppression mediated by the interaction of exosomal PD-L1 secreted by tumor cells with PD-1 expressed on the surface of activated T cells is of wide interest to researchers (34, 35). Activation of anti-tumor immune responses occurs when exosomal PD-L1 is blocked using antibodies against PD-L1. Therefore, targeting exosomal PD-L1 presents a new opportunity for enhancing existing immunotherapy.

## What important role does exosomal PD-L1 play in tumor immunity?

The role of exosomal PD-L1 in facilitating immune evasion in lung cancer is of paramount importance, as it impacts the immune system through various mechanisms. These encompass the suppression of T cell activity, modulation of other immune cell populations, and facilitation of an immunosuppressive microenvironment. Comprehending these intricate mechanisms is imperative for the advancement of novel therapeutic approaches aimed at counteracting immune evasion in lung cancer.

### Inhibition of T cell function

Tumor cells employ a strategy to evade immune surveillance by increasing the presence of PD-L1 on their surface. This protein interacts with PD-1 on T cells responsible for fighting against tumors, leading to the suppression of the anti-tumor response of T cells. Experiments conducted on cells revealed that exosomal PD-L1 exhibits identical membrane topology to cell surface PD-L1 and exerts comparable inhibition on T cells (36–38). Exosomal PD-L1 impedes the stimulation of T cells facilitated by the TCR. The binding of exosomal PD-L1 to PD-1 leads to a structural alteration in PD-1, initiating the phosphorylation of the immunoreceptor tyrosine inhibitory motif (ITIM) and the immunoreceptor tyrosine motif (ITSM). The phosphorylation subsequently results in the enlistment of cytoplasmic protein tyrosine phosphatases SHP-1 and SHP-2. As a result, SHP-1/2 hinders the internal phosphorylation of PI3K/AKT/mTOR, leading to the eventual cessation of T cell activation (**Figure 2D**). PD-1 functions by suppressing T cell activation through the inhibition of TCR signaling, which necessitates the engagement of TCR and co-stimulatory receptors with major histocompatibility complex (MHC) molecules, the interaction between MHC-I and TCR enhances the inhibitory function of exosomal PD-L1 on T cell (39, 40). Additionally, studies have shown that exosomal PD-L1 can effectively impede the phosphorylation of ERK and activation of nuclear factor kappa-B (NF-κB) in T cells induced by CD3/CD28 in a dose-dependent fashion, thus exhibiting immunosuppressive characteristics (41).

Significantly, in clinical data, the immunosuppressive impacts of exosomal PD-L1 often show a correlation with the levels of exosomes (42, 43). Kim et al. discovered that solely PD-L1^++++high^ exosomes suppressed the generation of pro-inflammatory cytokines, including Interleukin 2 (IL-2) and IFN-γ, in patients with non-small cell lung cancer (NSCLC) (12). Prolonged exposure to elevated levels of exosomal PD-L1 can result in the T cells experiencing a state of ‘functional exhaustion’. This depleted state affects the long-term response of the immune system and reduces its ability to respond to tumors. Nonetheless, the immunosuppressive phenotype cannot be definitively attributed to TEX alone due to the presence of a mixture of TEX and exosomes derived from non-malignant cells. Further onwards, a method based on immunoaffinity was employed to differentiate between exosomes derived from melanoma cells (MTEX) and exosomes derived from normal cells (non-MTEX) (21). Whiteside et al. found that MTEX itself was able to downregulate CD69 expression and inhibit CD8+ T cell proliferation in a PD-1 dependent manner. This discovery additionally indicates that exosomes originating from tumors predominantly contribute to the suppression of the immune system. In general, the quantities of exosomal PD-L1 might indicate a constantly changing relationship between cancerous and immune cells. Exosomes are capable of inducing an immunosuppressive phenotype by directly linking to PD-1, to inhibit the function of cytotoxic T cells and enable tumor immune escape.

### Impact on other immune cell types

Exosomes have been discovered to induce the expression of PD-L1 on secondary cells, such as macrophages and dendritic cells (DCs). This leads to the transfer of functional PD-L1 between cells and the creation of systemic immunosuppressive microenvironments, ultimately aiding in the facilitation of metastasis (44, 45). Liu et al. discovered that stress in the endoplasmic reticulum generates exosomes derived from hepatocellular carcinoma cells. These exosomes contain abundant microRNA (miRNA/miR)-23a-3p, which has the ability to enhance PD-L1 expression in macrophages via the phosphatase and tensin homolog (PTEN)/AKT pathway. Consequently, this leads to the inhibition of T cell immune function (46). Anti-PD-L1 antibodies rescue the immunosuppressive effect caused by exosomes from Lewis lung carcinoma cancer cells, which upregulate PD-L1 expression on DCs to hinder the proliferation of CD4+ T cells (47). In addition, Wei et al. discovered that exosomal PD-L1 derived from squamous cell carcinoma of the head and neck stimulates the development of activated regulatory T cells and M2 type macrophages. This, in turn, strengthens the creation of a positive feedback loop, hinders T cell growth, and fosters the development of a tumor immunosuppressive micro-environment (48). MDSCs are a heterogeneous group of cells known for their potent immunosuppressive functions. Exosomal PD-L1 may promote the proliferation and activation of MDSCs, enhancing their ability to suppress immune responses. Activated MDSCs inhibit T and natural killer cell (NK) cell functions by producing immunosuppressive molecules like arginase and nitric oxide synthase, further weakening the immune attack on tumor cells (49, 50). Interaction between exosomal PD-L1 and various immune cell types reduces the activity of these cells, thereby diminishing the overall anti-tumor immune response.

### Promotion of an immunosuppressive microenvironment

Exosomal PD-L1 plays a significant role in promoting an immunosuppressive microenvironment, a key factor in cancer progression and immune evasion, particularly in lung cancer. Exosomal PD-L1 promotion of the tumor immunosuppressive microenvironment is a complex process involving multiple immune cell types and signaling molecules. The promotion of immunosuppressive tumor microenvironment (TME) formation by exosomal PD-L1 through termination of T cell activation and maintenance of T cell depletion has been confirmed by numerous studies. In addition, exosomal PD-L1 has the ability to greatly impact the TME and facilitate immune evasion and tumor advancement by controlling the generation and release of cytokines and chemokines. The release of exosomal PD-L1 was increased in melanoma and glioblastoma cells, possibly due to the induction of cytokines such as IFN-α, IFN-γ, and tumor necrosis factor-α (TNF-α) (51, 52). In the xenograft mouse model of oral squamous cell carcinoma, mitochondrial Lon-induced exosomal PD-L1 promotes T cell dysfunction and tumor progression by inducing IFN and IL-6 production in M2 macrophages (53). Exosomal PD-L1 modifies the equilibrium of cytokines and chemokines, thereby establishing a milieu that inhibits the immune response against tumors, consequently promoting the survival and growth of tumor cells during immune surveillance.

In conclusion, exosomal PD-L1 plays an important role in promoting immune evasion in lung cancer by impeding effective responses to antitumor therapy. Exosomal PD-L1 exhibits increased resistance to protein hydrolase degradation, potentially leading to a heightened immunomodulatory function within the bloodstream and tumor microenvironment (54). Increasing evidence has shown that exosomes play a crucial role in promoting immune evasion through PD-L1, with a specific emphasis on the connection between exosomal PD-L1 and treatment response. Furthermore, this implies that the identification of exosomal PD-L1 holds promise in the early detection of diseases and the assessment of tumor prognosis.

## What is the novel discovery of exosomal PD-L1 in the diagnostic and prognostic applications of lung cancer?

Lung cancer consists of different cell populations with varying molecular alterations, leading to heterogeneity of the tumor and microenvironment (55). Indeed, the targeted alterations that initially predominate become less abundant during the course of the disease due to the selection of drug resistant sub-clones. In pursuit of finding biomarkers with sufficient sensitivity and specificity for early diagnosis and close monitoring of diseases, to help select optimal therapies and enable personalized medicine, it is crucial to identify these molecular signatures during the evolution of the disease.

Exosomes have the ability to serve as non-intrusive biomarkers that can enhance or add to the conventional biopsy, as they carry the characteristics of their parent cells. The membrane of exosomes protects their contents, which are present in different physiological fluids, from oxidation during transport (56). Akbar et al. have shown that exosomal PD-L1 is present in every individual diagnosed with NSCLC, while tissue PD-L1 is expressed in only 71% of patients. This implies that exosomal PD-L1 serves as a stronger indicator for diagnosis (57). Simultaneously, Ricklefs et al. investigated the relationship between exosomal PD-L1 immunohistochemical traits and clinicopathological aspects. They found that exosomal PD-L1 levels were elevated in NSCLC patients, especially in advanced stages, compared to healthy controls (51). Hence, the presence of exosomal PD-L1 in the bloodstream could serve as a promising indicator for the detection of tumors.

Concurrently, exosomal PD-L1 has been extensively investigated as a marker for forecasting the efficacy of immunotherapy and tracking the advancement of cancer in patients. The primary tumor load in mice with tumors was significantly reduced when treated with GW4869, an inhibitor of exosome secretion, and PD-L1 antibody. This indicates a synergistic connection between diminishing exosomes and immune checkpoint therapy in BALB/c 4T-1 tumor-bearing mice (41). Chen et al. discovered that the initial levels of exosomal PD-L1 were notably reduced in melanoma patients who exhibited a positive response to treatment using an anti-PD-1 medication (pembrolizumab) compared to those who did not respond (58). Clinicians can assess exosomal PD-L1 levels prior to treatment to identify patients who are likely to benefit from PD-1/PD-L1 inhibitor therapy and modify treatment approaches accordingly. A different research team investigating the correlation between exosomal PD-L1 and the effectiveness of immunotherapy discovered that treatment responsive patients had significantly reduced levels of PD-L1 in plasma-derived exosomes, while patients with disease progression had higher levels. However, no notable alterations were observed in patients with stable disease (SD) (59). Among patients with NSCLC, a study with comparable findings revealed that the expression of PD-L1 in exosomes was linked to the size of the tumor, the status of lymph nodes, the occurrence of metastasis, and the progression of the tumor (60). All the aforementioned discoveries indicate the possibility of identifying exosomal PD-L1 and its correlation with the effectiveness of immunotherapy.

In general, the growing body of evidence indicates that exosomal PD-L1 has the potential to be a new and reliable biomarker suitable for disease diagnosis as well as prognostic assessment (**Table 1**) (12, 25, 60–66).

**Table 1:** Clinical evidence of exosomal PD-L1 serving as a biomarker in lung cancer.

Exosome source	Detection methods	Clinical significance	References
Plasma	Flow cytometry and Immunohistochemistry	PD‐L1 abundance in exosomes correlated with PD‐L1 positivity in tumor tissues	(12)
Serum	Total exosome isolation kit, Western blot, TEM analysis, NTA	Higher exosomal PD‐L1, presented in patients with advanced tumor stage, larger tumor size, positive lymph node status and distant metastasis. Higher exosomal PD‐L1 presented in patients than normal controls	(25)
Serum	Western blot, TEM analysis, NTA, ELISA, Immunohistochemistry	Exosomal PD-L1 levels were higher in NSCLC patients with advanced tumor stage, larger tumor size (> 2.5 cm), positive lymph node status and distant metastasis	(60)
Serum	Surface plasmon resonance	Higher exosomal PD‐L1 presented in patients than normal controls	(61)
Plasma	Exosome isolation kit, Immunohistochemistry, NTA, TEM analysis, Western blot	The combination of blood PD-L1 mRNA and exosomal PD‐L1 could better determine NSCLC patients who may benefit from ICIs treatment	(62)
Plasma	NTA, TEM analysis, Western blot	Patients with exosomal PD‐L1 decrease tend to experience longer PFS than those with increasing levels	(63)
Plasma	Western blot, TEM analysis, ELISA, Immunohistochemistry	Lung cancer patients had much expression of PD-L1 in blood exosomes and high exosomal PD‐L1 content was linked to positive lymph node status in lung cancer patients	(64)
Plasma	TEM analysis, NFCM, Confocal fluorescence microscopy, Western blot	SCLC patients with tumor progression exhibited an increase in circulating exosomal PD‐L1 levels during treatment	(65)
Plasma	NTA, TEM analysis, Flow cytometry	Increased PD-L1^+^ exosomes are associated with a six-fold increased risk of disease progression in NSCLC patients.	(66)

TEM, transmission electron microscope; NTA, nanoparticle tracking analysis; PD-L1, programmed cell death ligand-1; ELISA, enzyme-linked immunosorbent assay; NSCLC, non-small cell lung cancer; ICIs, immune checkpoint inhibitors; PFS, progression-free survival; NFCM, nano-flow cytometer; SCLC, small cell lung cancer.

## How to target exosomal PD-L1 to optimize anti-tumor therapy strategies in lung cancer?

Exosome secretion relies heavily on nSMase2 and Rab27a, which play crucial roles in the budding of intravesicular vesicles and the fusion of multivesicular bodies with the plasma membrane. The release of exosomal PD-L1 was inhibited in cancer cell lines when nSMase2 and Rab27a were experimentally knocked down (17). As previously stated, blocking the release of PD-L1 through exosomes partially reestablishes the immune system’s ability to fight against tumors. In this particular situation, hindrance of exosomal PD-L1 secretion or obstruction of exosomal activity appears to be a promising treatment objective that may improve traditional therapeutic strategies.

Confirmation has been received that the removal of nSMase2 leads to a reduction in levels of PD-L1 in exosomes by hindering the synthesis of exosomes containing PD-L1. Compared with nSMase2 deletion, Rab27a deletion had a greater inhibitory effect on exosome secretion. Blocking Rab27a or nSMase2 can inhibit tumor growth (67). Both the deletion of Rab27a and the inhibition of nSMase2 (using GW4869) in the mouse model of drug-resistant breast cancer can effectively suppress tumor growth, surpassing the inhibitory impact of the anti-PD-L1 antibody at 30 and 60 units (41, 68). Interestingly, when mice were injected with mutant cancer cells that did not have Rab27a, nSMase2, or PD-L1, not only did the local tumor tissue fail to grow, but it also prevented the growth of wild-type tumor cells that were injected at the same time or 92 days later. In addition, when comparing wild-type cancer cells alone, there was a notable increase in both the quantity and functionality of exosomal PD-L1 in the tumor tissue of mice co-injected with tumor infiltrating lymphocytes (TILs) deletion mutant cells. This suggests that blocking the exosomal PD-L1 gene can effectively trigger a persistent systemic immune response (17). Clearly, the suppression of exosomal PD-L1 generation and release has arisen as a fresh and important strategy for the advancement of anticancer medication.

Several studies have demonstrated that the release of exosomes can be hindered through the use of antibodies, chemical inhibitors, or genetic manipulation, thereby enhancing the effectiveness of metastatic cancer treatment (69–71). Dimethyl amphotericin (DMA) hinders the release of exosomes by specifically targeting H/Na and Na/Ca^+++^2^+^ channels. This action eliminates exosome-induced immunosuppression and boosts the body’s ability to fight against tumors, making it an effective chemotherapeutic treatment (72, 73). The process of exosome formation, cargo allocation, and secretion is largely dependent on the ESCRT mechanism. Several pharmacologically active substances, including tipifarnib, combazole, triademenol, manumycin A, and nexinhibs, have been found to decrease the expression of proteins that are crucial in ESCRT dependent exosome biogenesis and transport, thereby exhibiting inhibitory effects on exosomes (74–76). Furthermore, the secretion of exosomes is blocked by GW4869 and spiro epoxide, which are inhibitors of nSMase independent of ESCTR (77, 78). Researchers have explored the extraction of exosomes from the bloodstream using *in vitro* ultrafiltration as a means to impede the proliferation of cancer cells. One benefit of this method compared to chemical medications is the ability to prevent harmful effects on healthy cells and potential drug interactions (79, 80). Overall, exosomal PD-L1 depletion may be an efficient adjunct therapy to alleviate systemic immunosuppression.

Nevertheless, caution should be exercised in the progress of exosomal PD-L1 inhibitors for anti-cancer treatment, as exosomes have a significant impact on numerous physiological processes within the human body, and disrupting their release could lead to potential negative consequences. In addition, similar to ICIs, the recovery of T cell activation mediated by exosomal PD-L1 is non-specific and may lead to immune related adverse effects (irAEs). To optimize the immune response against tumors and minimize potential side effects from inhibiting the release of exosomal PD-L1, it is crucial to create specific inhibitors of exosomes that selectively target cancer cells (81, 82). The exploitation of exosomes inhibitors as novel anticancer therapeutic agents has important implications for immunotherapeutic approaches to cancer treatment.

Besides serving as a crucial medium for intercellular communication, exosomes play a vital role in identifying responsive cancer patients and predicting treatment outcomes, in addition to indicating treatment response through targeted exosomal PD-L1 levels. Exosomes have been extensively investigated as a liquid biopsy and a reliable substitute for tumor tissue biopsy. However, the possibility of using exosomes as predictive screening tools for clinical applications is limited by the lack of harmonized exosome isolation procedures, appropriate quality control, and storage methods. Methods such as ultracentrifugation, precipitation, and size exclusion chromatography are some of the traditional methods, while microfluidic-based separation technologies, including nanoplatforms, have recently been investigated for the development of next-generation efficient separation methods (**Table 2**) (83–96). There is no doubt that when these challenges are addressed, exosomes are likely to be instrumental in the treatment of lung cancer.

**Table 2:** The advantages and disadvantages of techniques being used for the isolation of exosomes.

Iisolation method	Isolation techniques	Working principle	Advantages	Disadvantages	References
Centrifuge	Ultracentrifugation	The sedimentation coefficients of particles in the samples are different, which causes them to precipitate in different layers that can be collected separately	Easy to use, easy to operate and does not require complex sample pretreatment	Time-consuming and low purity of exocrine body	(83, 84)
Precipitate	Precipitation	Exosomes are added to the solvent to change the solubility of exosomes, causing them to precipitate from the solution	Large amounts of samples can be processed, easy to use	Pre- and post-cleanup are required, lower efficiency of isolation	(85, 86)
Capture	Immunoaffinity-based capture	Extraction of exocrine bodies according to the interaction between surface biomarkers (antigens) and immobilized antibodies	High recovery rate and high purity	Complex, special equipment, high cost	(87, 88)
Size-based isolation	Size exclusion chromatography	Polymer chromatographic column filled with anisotropic porosity	Isolation without the presence of albumin in purified exosomes	Low recovery and purity	(89, 90)
	Ultrafiltration	Size difference	Many samples can be processed simultaneously	Sample loss, vesicle deformation	(91, 92)
Microfluidics	Microfluidics-based	Isolation with miniaturized devices in various approaches such as acoustic, dielectrophoresis, filtration	Large amounts of samples can be processed, easy to use	Low isolation capacity, lack of global protocols and standardization, and high technical expertise are required	(93, 94)
Charge-based isolation	Charge-based isolation	The exocrine with a negative charge on the surface and carrying charge in electrophoresis can be separated under an electric field.	High efficiency and purity	There are higher requirements for sample types.	(95, 96)

## Future perspectives, challenges, and conclusion

Exosomes, being the main vehicles for cellular content transfer, have garnered interest due to their ability to modulate the immune system. Current research focuses on investigating the function of exosomes in cancer immunity and the response to immunotherapy, stemming from the established link between TEX and decreased immune function and immunotherapeutic efficacy.

In summary, the exosomal PD-L1 expression level shows a positive association with tumor stage and disease advancement, while exhibiting a negative correlation with survival. PD-L1, sourced from TEX, has the capability to directly interact with and impede the functional activity of T cells. Furthermore, the utilization of both anti-PD-1 treatment and exosome elimination resulted in a decrease in tumor load and enhanced overall survival. The results provide strong evidence that exosomal PD-L1 plays a crucial role in facilitating tumor development and spread, as well as suppressing the immune system. Current immunotherapeutic approaches solely focus on inhibiting PD-1/PD-L1 present on the outer layer of cancerous cells. However, cancerous cells additionally secrete exosomal PD-L1, which avoids the immune system’s reaction by attaching to PD-1 on T lymphocytes and impeding the activation of CD8+ T cells. Hence, the utilization of exosomal PD-L1 inhibitors in anti-exosomal PD-L1 therapies can induce robust systemic anti-cancer immunity and conquer the resistance to current anti-PD-1/PD-L1 treatments. Nevertheless, there is an inadequate number of preclinical investigations and/or clinical trials assessing the therapeutic capacity of exosomes in lung cancer, and the majority of these studies are retrospective with limited cohort sizes. Hence, further extensive investigations are required to validate if supplementary treatments that hinder the release of exosomes can enhance existing immunotherapy approaches.

A growing body of research is concentrating on the utilization of exosomal PD-L1 as an indicator for predicting the effectiveness of immunotherapy. Exosomes derived from tumors carry substances that induce cell release and can be detected in the blood, making them valuable non-invasive biomarkers that can enhance or supplement traditional biopsy procedures. Equally important is the need for improved methods of exosomes isolation and purification to study exosomes composition, characterization, and cellular interactions, which will set the foundations for their therapeutic applications.

This paper provides a summary of the suppressive effects of exosomal PD-L1 in lung cancer and its potential as a marker for early cancer detection, tumor advancement, and immunotherapy targeted at tumors. However, many questions are still unanswered. The complexity of the immune escape mechanisms of tumor cells involves multiple cell types and signaling pathways, including exosomal PD-L1, which poses a challenge to develop and improve therapies specifically targeting exosomal PD-L1. Moreover, although exosomal PD-L1 is considered a significant biomarker for tumor diagnosis and prognosis, there are still challenges in its detection accuracy and clinical relevance. How to develop uniform isolation and assay standards to identify exosomes to differentiate between TEX and non-malignant cell sources for better clinical application is an urgent issue. Immunotherapy targeting the PD-1/PD-L1 pathway may be resistant in some cases, which limits the efficacy of treatment. Clinicians are also concerned about the anxiety caused by the negative impacts of non-discriminatory exosomal PD-L1 inhibitors on individuals with cancer. Addressing these inquiries will aid in the swift enhancement and advancement of exosomal PD-L1 as a prognostic screening instrument for medical application, thereby augmenting the probability of cancer patients achieving a more efficacious and enduring reaction to immunotherapy. Further research should delve deeper into comprehending the precise function of exosomal PD-L1 in evading the immune system by tumors. By combining immunotherapy that targets exosomal PD-L1 with conventional treatments like chemotherapy and radiotherapy, it is possible to achieve a synergistic outcome and enhance the treatment’s success rate. Enhancing the identification technique of exosomal PD-L1, augmenting its accuracy and precision, and customizing individualized treatment plans according to patient specific tumor attributes and exosomal PD-L1 expression may enable a more precise evaluation of tumor characteristics and therapeutic effectiveness. By gaining a deeper comprehension of tumor biology, it becomes possible to create new medications and treatments, particularly specialized inhibitors or antagonists that focus on exosomal PD-L1.

To summarize, exosomal PD-L1 holds immense promise for its clinical utility as a novel focal point and biomarker in immunotherapy. However, it encounters various obstacles that necessitate resolution through ongoing research and inventive approaches.

## Author contributions

YuW: Writing – review & editing, Writing – original draft. HF: Writing – review & editing, Writing – original draft. JH: Writing – review & editing, Visualization, Validation, Methodology, Investigation, Data curation, Conceptualization. ZY: Writing – review & editing, Visualization, Validation, Methodology, Investigation, Data curation, Conceptualization. XQ: Writing – review & editing, Visualization, Validation, Methodology, Investigation, Data curation, Conceptualization. YL: Writing – review & editing, Visualization, Validation, Methodology, Investigation, Data curation, Conceptualization. RZ: Writing – review & editing, Visualization, Validation, Methodology, Investigation, Data curation, Conceptualization. TL: Writing – review & editing. YiW: Writing – review & editing. MW: Writing – review & editing.
